# Association of supply sources of alcohol and alcohol-related harms in adolescent drinkers: the baseline characteristics of a high school cohort across Thailand

**DOI:** 10.1186/s12889-022-14767-5

**Published:** 2022-12-05

**Authors:** Jirada Prasartpornsirichoke, Rasmon Kalayasiri, Polathep Vichitkunakorn, Woraphat Ratta-apha, Wanlop Atsariyasing, Natwarat Anekwit, Warot Lamyai, Chanchai Thongpanich, Surinporn Likhitsathian, Teerayuth Rungnirundorn, Wanida Rattanasumawong, Nawapat Chuatai, Sakol Srisuklorm, Athip Tanaree, Roengrudee Patanavanich

**Affiliations:** 1 Department of Psychiatry, Faculty of Medicine, Chulalongkorn University, 1873 Rama 4 Road, Pathumwan, Bangkok, 10330 Thailand; 2 Department of Psychiatry, King Chulalongkorn Memorial Hospital, Bangkok, Thailand; 3 Department of Family and Preventive Medicine, Faculty of Medicine, Prince of Songkla University, Songkla, Thailand; 4 Department of Psychiatry, Faculty of Medicine Siriraj Hospital, Mahidol University, Bangkok, Thailand; 5 Department of Mental Health, Psychiatry and Drugs, Mahasarakham Hospital, Mahasarakham, Thailand; 6 Nakhon Phanom Rajanagarindra Psychiatric Hospital, Nakhon Phanom, Thailand; 7 Thanyarak Khon Kaen Hospital, Khon Kaen, Thailand; 8 Department of Psychiatry, Faculty of Medicine, Chiang Mai University, Chiang Mai, Thailand; 9 Department of Psychiatry and Neurology, Phramongkutklao Hospital, Bangkok, Thailand; 10 Songkhla Rajanagarindra Psychiatric Hospital, Songkhla, Thailand; 11 Department of Community Medicine, Faculty of Medicine Ramathibodi Hospital, Mahidol University, Bangkok, Thailand

**Keywords:** Alcohol drinking, Alcohol supply, Alcohol–related harm, Alcohol use disorder, Underage drinking

## Abstract

**Background:**

The main objective of this study was to investigate the association between parental supply of alcohol, alcohol–related harms, and the severity of alcohol use disorder in Thai 7th grade middle school students.

**Methods:**

A cross–sectional descriptive study obtained the baseline data from the project named the Thailand Parental Supply and Use of Alcohol, Cigarettes & Drugs Longitudinal Study Cohort in Secondary School Students in 2018. The sample size was 1187 students who have ever sipped or drank alcohol in the past 12 months. Pearson’s Chi square, binary logistic regression, and ordinal logistic regression are applied in the analysis.

**Results:**

A single source of parental supply is not significantly associated with any alcohol-related harm and the severity of alcohol use disorder, while parental supply with peers and siblings supply of alcohol plays an important role in both outcomes. The increasing number of sources of alcohol supply increases the risk of alcohol–related harm and the severity of alcohol use disorder. Other risk factors found in both associations included binge drinking, alcohol flushing, low household economic status, distance from the student’s family, and poor academic performance. Gender, exposure to alcohol ads on social media and location of residency were not associated with alcohol–related harms or severity of alcohol use disorder.

**Conclusions:**

The results did not support parental guidance in teaching or giving children a drink or sip of alcohol within family to prevent related harms when drinking outside with their peers.

## Introduction

The trend of alcohol consumption in Thai adolescents has increased considerably in the last decade. According to the Centre for Alcohol Studies of Thailand, the prevalence of alcohol consumption among high school students increased from 19% in 2007 to 26% in 2017 [1]. Early alcohol intake, particularly before the age of 15, can predispose adolescents to alcohol-related problems, as well as poor adult outcomes [2–4]. However, when other individual and environmental variables are well controlled, the impact of early alcohol exposure; initiation of an adolescent’s own drinking, on adult outcomes could be reduced [3]. Previous research in Thailand found that the average age of first exposure to alcohol among grade 10 students in Nan province, Northern Thailand, was 14.5 years, with the lowest age of 9 years [5]. In addition, among the sample of female students in high schools in the Eastern province of Thailand, more than one third of female drinkers had their first alcohol consumption before high school, and 15.6% had first been exposed when they were younger than 10 years old [6].

In Thailand, it is against the law for retailers to sell alcoholic beverages without required licenses [7], and anyone –including parents, relatives, and caregivers –is prohibited from giving an alcoholic beverage to a child or adolescent under the age of 20 [8] and 18 years old [9]. Many Thai parents, on the other hand, become the suppliers to their children with the belief of protecting them from alcohol–related harms and alcohol use disorders when their reach adulthood, especially in the case of daughters. In addition to previous literature in Australia and Canada reporting that parents were a major source of alcohol supply to adolescents [10, 11] and were found to be a protective factor for adolescent alcohol consumption [10, 12]. On the contrary, recent research found that adolescents who had their parents provide them with alcohol had a higher risk of binge drinking, alcohol–related harm, alcohol use disorder, and other alcohol outcomes than those who did not have their parents provide them with alcohol [13–17].

Not only the parental supply of alcohol, but also other supplies of alcohol play the important role in the outcome of alcohol outcome. Previous studies found a higher degree of impact of peer supply on adolescent alcohol use and outcomes [12, 14, 18]. However, increasing sources of alcohol supply in adolescents did not guarantee a higher probability of alcohol outcomes. Mattick et al. (2018) found that the odd ratios of two sources of alcohol supply; parents and others, in binge drinking and alcohol abuse were less than one source of alcohol supply, especially from non-parental sources, while the odd ratios of two sources of alcohol supply on alcohol-related harms, alcohol dependence and alcohol use disorder were definitely higher than one source of alcohol supply [14].

Other factors influence the effects of adolescent alcohol use in addition to the sources of alcohol supply. Previous studies have found that drinking alcohol is influenced by social and physical variables. Regarding social issues, the student’s values or behavior, societal standards, school-related variables, and liquor advertising can all have an impact on a minor’s behavior and alcohol use [19]. In terms of a physical variable, earlier research discovered that the alcohol flush reaction, an aversive reaction when drinking alcohol including erythema of the face, nausea, and palpitation caused by the deficiency of enzyme aldehyde dehydrogenase (ALDH), increased the rate of occurrence of harm to health in alcohol drinkers although decreased harmful alcohol use, such as dependence, abuse, maximum drink, or binge drinking over the course of a lifetime [20–22].

The objective of the study was to investigate whether different sources of alcohol supply were associated with different experiences of alcohol-related harm and differing severity of alcohol use disorder in Thai 7th grade middle school students. For the purpose of controlling confounding effects, there are additional socioeconomic, behavioral, and physical variables in the model. These variables included household income, media exposure to alcohol advertising, biological responses to alcohol, and academic performance.

## Methods

### Procedure

This cross–sectional descriptive study obtained data from the Thailand Parental Supply and Use of Alcohol, Cigarettes, and Drugs Longitudinal Study Cohort in Secondary School Students survey, which collected data in 2018/2019 from Thai seventh grade students aged 12–15 years and their closest parents or guardians. The questionnaires were distributed to students and their parents by members of the research team at five different locations throughout Thailand. The questionnaires had to be filled out by both students and parents, and all forms had to be returned to be included in the study. Consent was obtained by action (that is, return of forms). This survey project was conducted in accordance with the Declaration of Helsinki and has been approved by the Institutional Review Board (IRB) of the Faculty of Medicine, Chulalongkorn University. Informed consent was obtained from all subjects and their legal guardians. The IRB number is 590/61.

**Fig. 1:**
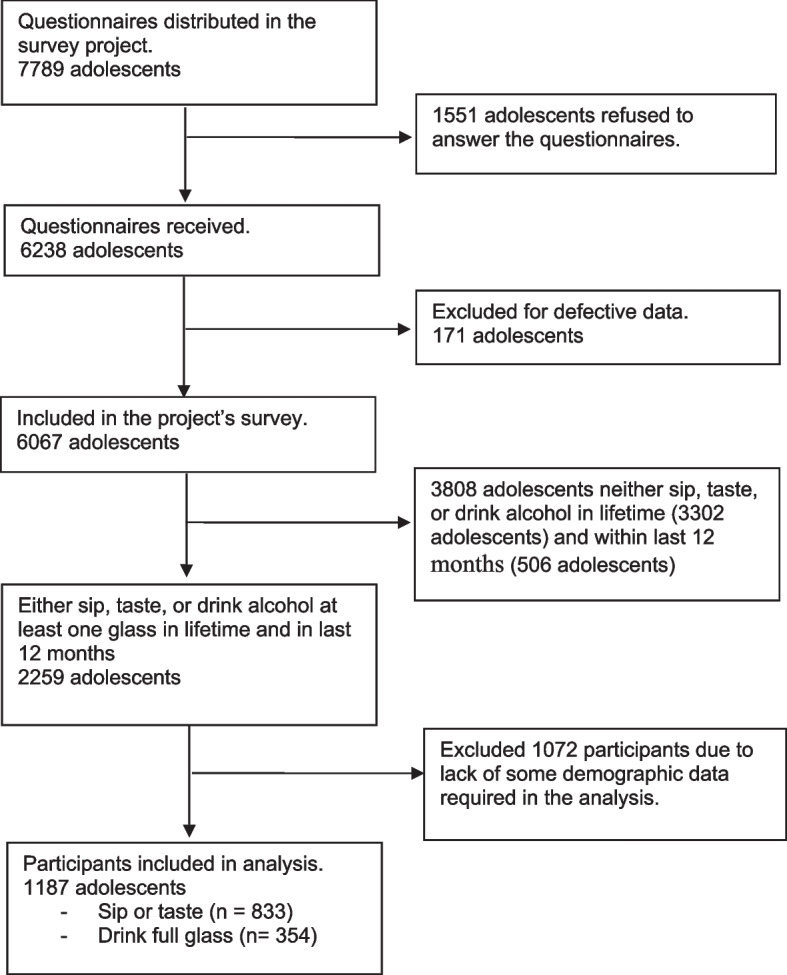
The flow diagram of the participants recruited in the study

The study included 7789 seventh grade Thai students and their parents from three types of schools: municipal public schools, non–municipal public schools, and independent (private) schools, all of whom lived in five different areas and regions of Thailand: Northern, Northeastern, Central, Southern, and Greater Bangkok. When collecting data and filling demographic information, only Thai teens who had ever tasted, sipped, fully drank, or binged alcohol in the previous 12 months were included in this study. As illustrated in Fig. 1 of the flow chart, the total sample size was 1187 participants.

### Measurement

#### Alcohol use disorder

Alcohol use disorder was assessed by 11 symptoms of the disorder identified in the fifth edition of the Diagnostic and Statistical Manual of Mental Disorders (DSM–5). The questionnaire was designed to examine the frequency of symptoms in the last 12 months of adolescents. Participants were determined to have a symptom occurrence of alcohol use disorder if they had experienced at least two symptoms of alcohol use disorder in the previous 12 months. In addition, there are three degrees of severity for alcohol use disorder: mild, moderate, and severe. Mild alcohol use disorder was defined as two to three symptoms in the previous 12 months, moderate was defined as four to five symptoms, and severe was defined as six or more symptoms in the previous 12 months [23].

#### Alcohol–related harms

Alcohol–related harms were evaluated over the previous 12 months using eight items related to alcohol intoxication and other negative effects of alcohol consumption, such as sexual harassment, getting drunk, legal difficulties, hangovers, and blackouts. This was adapted and based on questions from the Australian Parental Supply of Alcohol Longitudinal Study [14]. In this study, having any alcohol–related harms were measured as a dichotomous variable, with 0 indicating that no harm ever occurred and 1 indicating harms occurring during the previous 12 months.

#### Provision of alcohol by parents and other sources

This was also based on questions from the Australian Parental Supply of Alcohol Longitudinal Study [14]. In the preceding 12 months, adolescents were asked how often they had received alcohol from their parents (mother or father), relatives, siblings, or friends. The group of variables was modelled as dummy variables based on the person who supplied the alcohol and the number of sources of alcohol supply.

#### Perception of alcohol advertisement on social media

There were two main forms of alcohol advertisements on social media, according to the study: photographs and video clips. Thai middle school students were asked if they had ever watched a video or seen a photo on social media related to an alcohol commercial.

#### Alcohol response

Participants were asked to describe their alcohol flushing reaction, which included their face and palm turning hot and red in 2–3 minutes after drinking alcoholic beverage. Gene-controlled alcohol metabolism is strongly related to the alcohol flushing reaction.

#### Other socioeconomic and demographic factors

Gender, area of residence (given 1 if stay in Greater Bangkok and 0 if rural area), the most recent academic achievement (given 1 if the grade point average (GPA) was less than 2.5 and 0 if above 2.5), living status with parents and household economic sufficiency are all factors to consider. According to the National Statistical Office’s (NSO) survey, household income sufficiency was defined as the amount of income that was greater than the average household expenditures in 2019, which were 20,742 baht per month. All the variables given before were binary variables.

### Statistical analyzes

Descriptive data were presented as frequencies and percentages. We investigate the characteristics related to alcohol-related harms and alcohol use disorder using Pearson’s chi-square tests. Associations of parental alcohol supply and other relevant factors with alcohol-related harms and the severity of alcohol use disorder were examined using binary logistic regression (enter method) and ordinal logistic regression. Potential associated variables; the sources of alcohol supply and confounders; alcohol-drinking behavior, exposure to social networks, alcohol reaction, and other sociodemographic factors were included in the initial model. SPSS version 28.0 was used for our data analysis. A *P* value less than 0.05 was considered statistically significant.

## Results

### Sample characteristics and alcohol use

The characteristics of the respondents, the sources of alcohol supply, and outcomes such as alcohol–related harms and the severity of alcohol use disorder are listed in Table 1. Most of the respondents were female (*n* = 708, 59.6%), had a single source of alcohol supplies (*n* = 427, 35.9%), had no reaction of flushing with alcohol (*n* = 1093, 92.1%), had good academic performance (*n* = 1054, 88.9%) and had ever seen or watched alcohol advertisements in pictures or video clips while using social media platforms (*n* = 949, 78.7% and *n* = 982, 82.7%). Furthermore, 41.5% (*n* = 493) of Thai teenage drinkers received alcohol from their parents in the previous 12 months, and 22.2% of respondents (*n* = 263) and 22.0% of participants (*n* = 261) had experienced alcohol–related harms and alcohol use disorder, respectively.

**Table 1:** The number and percentage of sources of alcohol supply, alcohol response, exposure to alcohol advertising, alcohol-related harm, and alcohol use disorder in Thai junior high school students who drank alcohol (*n* = 1187)

Variables	n (%)
**Supply of alcohol**	
** 1. No supply from others (self**–**supply only)**	**293 (24.8)**
** 2. Single source of supply of alcohol**	**427 (35.9)**
- Parental supply only	170 (14.3)
- Friend/siblings supply only	163 (13.7)
- Relatives supply only	94 (7.9)
** 3. Two sources of supplies of alcohol**	**276 (23.2)**
- Parental and friend/siblings supply	73 (6.1)
- Parental and relatives supply	59 (5.0)
- Friend/siblings and relatives supply	144 (12.1)
** 4. More than two sources of supplies of alcohol**	**191 (16.1)**
- Parental, friend/siblings, and relatives supply	191 (16.1)
**Social media exposure to alcohol advertisements**	
- Exposure to a picture of alcohol advertisement	949 (79.7)
- Exposure to a video clip of alcohol advertisement	982 (82.7)
**Alcohol response**	
- Flushing reaction experience	94 (7.9)
**Socioeconomic and demographic variables**	
- Female	708 (59.6)
- Families with low income	439 (36.9)
- Not living with parents	188 (15.8)
- Living in Greater Bangkok	287 (24.2)
- Medium to low academic achievement (GPA less than or equal 2.5 (out of 4.0) or C+)	133 (11.1)
**Alcohol**–**drinking behavior**	
- Binge drinking two to three times per month	25 (2.1)
**Alcohol**–**related harms**	
- Any alcohol–related harms	263 (22.2)
**Alcohol Use Disorder**	
- None	926 (78.0)
- Alcohol Use Disorder	261 (22.0)
Mild alcohol use disorder	158 (13.3)
Moderate alcohol use disorder	64 (5.4)
Severe alcohol use disorder	39 (3.3)

Specific mild alcohol use disorder if there are two to three symptoms, moderate alcohol use disorder if there are four to five symptoms, and severe alcohol use disorder if there are six or more symptoms out of 11 symptoms at any time during the same 12-month period, according to the DSM-5 criteria

*GPA* Grade point average

Table 2 described the signs and symptoms of alcohol use disorder, as well as the items of alcohol–related harms that Thai seventh-grade middle school students had experienced in 12 months prior. According to descriptive data, students who observed any symptoms of alcohol use disorder drank more than they intended (*n* = 349, 29.4%) and craved alcohol the most (*n* = 238, 20.1%). Additionally, those who had experienced alcohol-related harm in the previous 12 months had the greatest difficulty getting drunk (*n* = 173, 14.6%).

**Table 2:** The proportion of Thai seventh-grade middle school students who drank alcohol in the previous 12 months experienced alcohol-related harms and alcohol use disorders. (*n* = 1187)

Alcohol use disorder	n (%)	Alcohol–related harms	n (%)
**No symptoms**	**723 (60.9)**	**No harm**	**924 (77.8)**
**Having at least one symptom**	**464 (39.1)**	**Harm (s)**	**263 (22.2)**
1. Drinking more than intended	349 (29.4)	1.Getting drunk	173 (14.6)
2.Trying / wanting to stop or cut down but unsuccessful	121 (10.2)	2. Planning to be drunk before drinking	80 (6.7)
3.Spending a lot of time searching, drinking, or recovering	164 (13.8)	3. Feeling uncomfortable after drinking	110 (9.3)
4.Craving for drinking	238 (20.1)	4.Having hang over	90 (7.6)
5.Having a problem at work, school, or family due to drinking	39 (3.3)	5.Having black out	60 (5.1)
6.Having a problem with others due to drinking	24 (2.0)	6.Having a problem with the police due to drinking	18 (1.5)
7. Reduce other social / recreational activities due to drinking	40 (3.4)	7.Having a regretful sexual relationship after drinking	7 (0.6)
8.Driving/doing activity that may cause accident when drinking	29 (2.4)	8.Being sexually harassed / abused when drinking	19 (1.6)
9.Having medical / physical / mental problems due to drinking	33 (2.8)		
10. Having tolerance for drinking	98 (8.3)		
11. Having alcohol withdrawal symptom	26 (2.2)		

If seventh grade middle school students encountered symptoms of alcohol use disorders and items of alcohol–related harms once in the previous year, they were counted

### The associations of alcohol supply and adolescent alcohol problems

The results of a bivariate analysis of alcohol supply, binge drinking, alcohol advertising exposure, alcohol reaction, other socioeconomic characteristics, and juvenile alcohol problems over the previous 12 months showed that having a single source of alcohol supply from a parent, the increased number of alcohol supply sources, binge drinking behavior, having an alcohol flushing reaction, having worse academic achievement, having a low household income, and not living with their parents were potential predictors significantly associated with alcohol–related harms (*P*-values < 0.01) and alcohol use disorder (*P*-values < 0.001) in 12 months prior.

The results of the binary logistic regression analysis investigating the impact of parental supply of alcohol and other factors on adolescent alcohol–related harm and alcohol use disorder are provided in Table 3. After controlling for the effect of gender, socioeconomic factors, location of living, and student academic performance in the model, we found that the single parental supply of alcohol was not associated with alcohol-related harms (adjusted odds ratio [AOR] = 1.066, 95%CI = 0.521–2.181, *p* = 0.861) and alcohol use disorder (AOR = 1.178, 95%CI = 0.606–2.294, *p* = 0.629). However, the alcohol provision of friends / siblings of the participants was significantly associated with any alcohol–related harms (AOR = 2.888, 95% CI = 1.636–5.098, *p* <  0.001) and alcohol use disorder (AOR = 2.664, 95% CI = 1.533–4.630, *p* <  0.001). Furthermore, the odds of experiencing alcohol-related harms and having alcohol use disorder increased by 6.357 times (95%CI = 3.764–10.735,* p* <  0.001) and 5.494 times (95%CI = 3.294–9.163, *p* <  0.001) if adolescents received alcohol from all sources; parents, friends/siblings, and relatives. Furthermore, binge drinking, alcohol flushing, low family income level, and not living with their parents were statistically significantly associated with alcohol-related harms and alcohol use disorder.

**Table 3:** Binary logistic regressions predicting alcohol–related harms and severity levels of alcohol use disorder in Thai adolescents (*n* = 1187)

Variables	Adjusted Odds Ratio: AOR (95% CI)	
	Any alcohol–related harms (1)	Alcohol use disorder (2)
**Supply of alcohol**		
Parental supply only (=1)	1.066 (0.521–2.181) *P* = 0.861	1.178 (0.606–2.294) *P* = 0.629
Friend/siblings supply only (=1)	**2.888 (1.636–5.098)** ***P*** **< 0.001**	**2.664 (1.533–4.630)** ***P*** **< 0.001**
Relatives supply only (=1)	**2.148 (1.061–4.350)** ***P*** **= 0.034**	0.775 (0.322–1.865) *P* = 0.569
Both parental and friend/siblings supply (=1)	**4.332 (2.211–8.488)** ***P*** **< 0.001**	**4.356 (2.259–8.400)** ***P*** **< 0.001**
Both parental and relatives supply (=1)	1.552 (0.641–3.759) *P* = 0.330	**3.184 (1.513–6.703)** ***P*** **= 0.002**
Both friend/siblings and relatives supply (=1)	**5.425 (3.094–9.511)** ***P*** **< 0.001**	**3.443 (1.963–6.038)** ***P*** **< 0.001**
All parental, friend/siblings, and relatives supply (=1)	**6.357 (3.764–10.735)** ***P*** **< 0.001**	**5.494 (3.294–9.163)** ***P*** **< 0.001**
**Alcohol-drinking behavior**		
Binge-drinking 2–3 times per month (=1)	**12.115 (3.881–37.823)** ***P*** **< 0.001**	**16.907 (4.817–59.345)** ***P*** **< 0.001**
**Social media exposure to alcohol advertisements**		
Picture (=1)	1.120 (0.626–2.006) *P* = 0.702	1.117 (0.626–1.990) *P* = 0.708
Video clip (=1)	1.407 (0.748–2.648) *P* = 0.290	1.001 (0.542–1.846) *P* = 0.998
**Alcohol reaction**		
Have alcohol flushing reaction (=1)	**3.915 (2.423–6.327)** ***P*** **< 0.001**	**3.292 (2.034–5.329)** ***P*** **< 0.001**
**Other sociodemographic factors**		
Female students (= 1)	0.991 (0.714–1.376) *P* = 0.957	1.069 (0.768–1.487) *P* = 0.694
Family economic status (total income less than average national expenditures =1)	**2.029 (1.470–2.802)** ***P*** **< 0.001**	**2.196 (1.592–3.029)** ***P*** **< 0.001**
Do not live with parents (=1)	**1.921 (1.305–2.828)** ***P*** **< 0.001**	**2.185 (1.488–3.209)** ***P*** **< 0.001**
Academic performance (GPA less than 2.5 = 1)	1.447 (0.921–2.273) *P* = 0.109	**1.887 (1.208–2.947)** ***P*** **= 0.005**
Live in Greater Bangkok (=1)	1.397 (0.973–2.008) *P* = 0.070	1.164 (0.805–1.684) *P* = 0.420

(1) using binary logistic regression model (Enter method), Nagelkerke R square = 0.269. The reference group is never facing any alcohol–related harm within 12 months prior

(2) using binary logistic regression model (Enter method), Nagelkerke R square = 0.268. The reference group is never having any symptoms of Alcohol Use Disorder within past 12 months

*CI* Confidence interval, *GPA* Grade point average

Table 4 explained the results of an ordered logistic regression model that examines the association of alcohol provision and other factors with the ordinal outcome named the severity of alcohol use disorder, which was divided into four categories: none (= 0), mild (=1), moderate (= 2) and severe (=3). In the regression, the reference group is the set of responses with fewer symptoms than the alcohol use disorder criteria (no alcohol use disorder). The results indicate that the single provision of alcohol from friends/siblings (AOR = 2.838, *p* <  0.001), the provisions of alcohol from two sources and greater (AOR = 5.433, *p* <  0.001), binge drinking behavior patterns (AOR = 12.057, *p* <  0.001), having an alcohol flush reaction (AOR = 3.151, *p* <  0.001), insufficient household income (AOR = 2.321, *p* <  0.001), staying separately from parents (AOR = 2.022, *p* <  0.001), and poor academic performance in the most recent semester (AOR = 1.920, *p* = 0.002) were statistically significant associated with a higher probability of higher severity levels of alcohol use disorder.

**Table 4:** Summarized statistics, threshold, coefficients, estimated odds ratio, and 95% confidence interval from ordinal logistic regression of the severity of alcohol use disorder

Variables	Coefficient	Wald Chi–squared	***p***–value	Adjusted Odds Ratio	95% Confidence Interval for Odds Ratio	
					Lower	Upper
**Threshold**						
No Alcohol Use disorder (=1)	3.007		< 0.001			
Mild Alcohol Use Disorder (=2)	4.329		< 0.001			
Moderate Alcohol Use Disorder (=3)	5.521		< 0.001			
	**Coefficient** **(β)**					
**Supply of alcohol**						
Parental supply only (=1)	0.179	0.281	0.596	1.197	0.616	2.322
Friend/siblings supply only (=1)	**1.043**	**14.265**	**< 0.001**	**2.838**	**1.652**	**4.877**
Relatives supply only (=1)	−0.286	0.411	0.521	0.751	0.313	1.803
Both parental and friend/siblings supply (=1)	**1.519**	**21.453**	**< 0.001**	**4.569**	**2.402**	**8.691**
Both parental and relatives supply (=1)	**1.004**	**7.309**	**0.007**	**2.730**	**1.318**	**5.654**
Both friend/siblings and relatives supply (=1)	**1.269**	**20.489**	**< 0.001**	**3.556**	**2.053**	**6.158**
All parental, friend/siblings, and relatives supply (=1)	**1.692**	**43.969**	**< 0.001**	**5.433**	**3.294**	**8.959**
**Alcohol**–**drinking behavior**						
Binge drinking 2–3 times per month (=1)	**2.490**	**42.184**	**< 0.001**	**12.057**	**5.688**	**25.558**
**Social media exposure to alcohol advertisements**						
Picture (=1)	0.149	0.269	0.604	1.161	0.661	2.038
Video clip (=1)	−0.035	0.013	0.908	0.966	0.534	1.745
**Alcohol reaction**						
Have alcohol flush reaction (=1)	**1.148**	**26.634**	**< 0.001**	**3.151**	**2.038**	**4.873**
**Other sociodemographic factors**						
Female students (= 1)	−0.047	0.084	0.772	0.954	0.694	1.312
Family economic status (total income less than average national expenditures =1)	**0.842**	**28.412**	**< 0.001**	**2.321**	**1.703**	**3.164**
Do not live with parents (=1)	**0.704**	**14.606**	**< 0.001**	**2.022**	**1.409**	**2.902**
Academic performance (GPA less than 2.5 = 1)	**0.652**	**9.162**	**0.002**	**1.920**	**1.259**	**2.930**
Live in Greater Bangkok (=1)	0.151	0.695	0.405	1.164	0.815	1.662

Significant level is at 0.05. Link function is cumulative Logit function. Model function is Multinomial distribution

*GPA* Grade pointed average

## Discussion

This cross–sectional study aimed to investigate the relationship between sources of alcohol and related factors, alcohol–related harms, and alcohol use disorders among 1187 Thai adolescents using the baseline characteristics of a middle school cohort in Thailand conducted in 2018. We found that among Thai teenagers, the prevalence of any alcohol–related harms and alcohol use disorders in the past 12 months was 22.2 and 22.0%, respectively. The major source of alcohol supply in this study was parents. This was consistent with the previous studies [24, 25]. After controlling for the effects of confounders, Thai seventh grade students who had ever sip or drank alcohol in the previous 12 months exhibited a significant influence of peer/sibling single supply of alcohol on both outcomes: any alcohol–related harms and alcohol use disorder. Although there was no statistically significant association between a single source of parental alcohol supply and both outcomes, increasing sources of alcohol provision escalated the probability of adolescents experiencing alcohol–related harms and the severity of their alcohol use disorder.

A single parental source of alcohol supply was found to be unrelated to any alcohol–related harms or the severity of alcohol use disorder. However, the result found that the parental or relative supply of alcohol with peers/siblings was significantly related to both outcomes in this study. The former was in line with some previous studies that found no causal association between parental alcohol supply and adolescent alcohol problems [26]. The insignificant relationship between parental supply of alcohol and alcohol outcomes in this study was explained for many plausible reasons. First, it was possibly because parents monitored, supervised, and limited alcohol consumption in adolescents [26]. Second, this was in part related to the analysis of the cross-sectional data. The previous literature found the significant adverse effect of parental, ever-sips or whole servers, on adolescent alcohol outcomes, including binge drinking, any alcohol-related harms, and symptoms of alcohol use disorder in the second to fifth waves of cohort observation, if compared with no supply in the first wave [14, 15, 27, 28]. This suggests that the single parental alcohol provision was neither a protective nor a risk factor for alcohol use issues in Thai adolescents. The East-West attitudes and norms would also be concerned about different results of the associations between parental supply of alcohol and alcohol outcomes. However, no empirical study was found in Asian countries.

However, alcohol supply was strongly linked to any alcohol–related harms and the severity of alcohol use disorder in Thai seventh-grade middle school students who had drank alcohol beverage in the previous 12 months. Peer influence had a major effect on adolescent drinking, according to previous research, due to friend selection, peer network dynamics, and unsupervised time with peers [19, 29, 30]. Adolescents who acquired alcohol from sources other than their parents were more likely to report alcohol–related problem behaviors, according to a previous worldwide study [18]. It is possible that unhealthy social networks, such as those surrounded by people who consume alcohol, could be a risk factor for adolescent alcohol use.

However, obtaining an alcohol beverage from two or more sources (eg, both parents and peers/siblings, or both relatives and peers/siblings) not only increased the odds of experiencing alcohol–related harm and the severity of alcohol use disorder, but also increased the odds with higher magnitude than obtaining alcohol from a single peer/siblings source. The result was consistent with previous evidence that found the greater odds ratio of alcohol outcomes when the adolescent obtained alcohol from the parents in addition to other sources [14, 18, 28, 31]. Parents could not limit alcohol consumption among their adolescents. This caused risky drinking or alcohol misuse among adolescents without supervision [28, 32]. The result confirmed the previous discussion that unhealthy social networks increased the greater risk caused by alcohol use among adolescents. Furthermore, if parents relinquish control of their children for a specific cause, such as the fact that the adolescent’s school is located far away from their house. This forces children and adolescents to live with relatives or in dormitories alone. If strict parental supervision is reduced, children and adolescents will have more opportunities to drink alcohol without parental supervision.

Insufficient family income was found to be significantly correlated with alcohol-related harms and the severity of alcohol use disorder. This was consistent with the results of previous studies, which found the significant association between low family income and adolescent alcohol use, alcohol drinking pattern, and alcohol problems [33–36]. On the other hand, some prior studies reported contradictory results on the effect of socioeconomic class on alcohol consumption. A high socioeconomic level was found to be substantially associated with increased teenage alcohol use through increased parental alcohol drinking and a change in high society perceptions of alcohol as a means of relaxation and stress release [37, 38]. The study also found significant relationships between middle schoolers’ poor academic performance and their both alcohol outcomes. The finding was consistent with prior research that identified low academic performance as a strong predictor of substance use, including smoking, alcohol, and drugs, among high school students [39, 40]. When studying at the undergraduate level, it was discovered that poor academic performance was not significantly associated with adolescent drinking habits [41]. This emphasized societal expectations that favored families with high socioeconomic standing and children who excelled at school. Adolescents with poor academic performance, especially those who were labeled “bad students” when they had low academic performance, tend to lose interest in the lessons that they are not good at and move their focus to other topics.

Other risk factors found in both associations included binge drinking and alcohol flushing reaction. The latter is in contrast with previous studies reporting flushing reaction as a protective factor against alcohol dependence or the maximum lifetime number of drinks within 24 hours [20, 39, 42]. Since this study is a cross–sectional study at a very early age of alcohol use, the protective effect of the flushing reaction may not be revealed until later in life. Furthermore, the flushing reaction after drinking alcohol might be viewed by teenage drinkers as an effect of alcohol that led them continue drinking. In contrast, students who did not have the reaction might stop or stop drinking, since they did not feel the alcohol effect regardless of whether the effect is aversive or rewarding.

However, sex, exposure to alcohol advertisements on social media, and location were not significantly related to alcohol-related harms or the severity of alcohol use disorder. Different from previous research that found the significant association between female and alcohol outcomes [12]. This is in part due to the fact that the respondents were between 13 and 15 years. Although exposure to alcohol advertisements did not associate with adolescents with alcohol use disorder or alcohol–related harms in this study, exposure could affect the initiation of alcohol as previously shown [43]. An additional analysis to include students who did not drink alcohol could show different results with respect to alcohol advertisement.

There were some limitations in this research. First, this was a cross–sectional study, it was insufficient to definitively determine the causal relationship between alcohol provisions and alcohol-related problems among Thai youth. Additionally, due to the lag time of juvenile alcohol problems, this cross-sectional study may underestimate the magnitude of the impact of parental and other alcohol provisioning on Thai adolescents’ alcohol problems. Second, this study excluded other behavioral risks, including drug use and smoking, and excluded the variability of number or frequency of alcohol supply from each source that could interact with alcohol consumption. This would be a space for future research. Third, recall bias could arise in the study, particularly when adolescents were asked about their alcohol consumption and problems in the previous 12 months. Finally, the limitations of the study include that the returns of the surveys from the parents and students were not separated. Bias of the data might occur when both know the answer. However, students are the persons who returned the survey back so the parents may not know the answers of the students but not vice versa (students may know the answer from the parents). Such that students would answer the survey more freely without being afraid that the parents may know their answers.

Our results did not support parental guidance in teaching or giving children a drink or sip of alcohol within family to prevent related harms when drinking outside with their peers. A longitudinal study to follow the students further would reveal more the effect of parental supply of alcohol and other related factors on alcohol–related harms and alcohol use disorder.

## Data Availability

The data sets generated during and/or analysed during the current study are available from the corresponding author on reasonable request.
